# A Treatise on the Anatomy, Physiology, and Pathology of the Centre of the Cerebro-Spinal Nervous System

**Published:** 1844-10

**Authors:** 


### ART. XII.

*Traité complet de l'Anatomie, de la Physiologie et de la Pathologie du Système nerveux Cerebro-spinal.* Par M. FOVILLE. 1re partie—*Anatomie.—Paris*, 18[illegible]4.

*A Treatise on the Anatomy, Physiology, and Pathology of the Centre of the Cerebro-spinal Nervous System.* By M. FOVILLE. First Part—*Anatomy.—Paris*, 1844. 8vo, pp. 676.

No portion of the human body requires such strict anatomical investigation as the brain and spinal cord; and in none has previous examination given greater difference in the result. We must therefore express our satisfaction at finding, in the still very imperfect state of our knowledge of the cerebro-spinal nervous centre, a book like M. Foville's, which attempts at least to add something to our stock of information.

Since the time of Reil, but few important additions have been made to his discoveries in the texture of the brain. The extreme difficulty of the inquiry deters many from entering on it; and it is the lot of but few to devote a sufficient number of years to the dissection of the hardened brain, to make their statements worthy of consideration. The author of the book before us has been occupied the greater part of his life with the study of the nervous centre; and he presents us, in this volume, a portion of the knowledge obtained from his labours, viz. the detailed anatomy of the brain and spinal cord in the adult, and the connexions of the nerves with that mass. Much as we approve of M. Foville's mode of proceeding, and greatly as we value his experience, we cannot consider some of his statements as altogether free from suspicion. To arrive at correct conclusions as to the structure and use of portions of the brain, we not only think that the aid afforded by comparative anatomy should be freely accepted, but we deem its aid almost essential. But M. Foville takes the opposite course, and considers it "necessary, before commencing those studies (of the lower classes), to be well acquainted with the knowledge of the most perfect type—that is to say, the nervous system of the adult man, in order to be able to make exact comparisons, and to arrive at correct conclusions." The correctness of such reasoning we consider very doubtful, and think the plan pursued likely to have led the author and to lead others into error. For it is in the lower classes that the complex forms of the higher are observed to be simplified, and as it were analysed; and difficulties that, at first sight, appear inexplicable, to be removed, by means of a series of successive steps, only gradually increasing in complexity as we ascend the scale of life. In the examination of the delicate structure of the brain, whose fibres are so interlaced that great judgment and dexterity are required in their separation, every assistance that may be obtained from other sources should be diligently sought, so as to leave no room for fallacy to creep in and invalidate the result. It is, unquestionably, much easier to rest satisfied with an examination of the human brain, than to repeat the same process in a great variety of animals, but much less satisfactory. In the case of the present author we cannot doubt that a more extended inquiry would have diminished the chances of hasty conclusion occasionally evident in this work. The author, for example, sometimes assumes that, because some fibres take a direction towards a certain spot, they most probably reach it. Such a supposition may doubtless contribute readily to solve some hitherto inexplicable theory; but if the disposition of those fibres were traced through simpler forms of development the examination might lead to very different and certainly much more trustworthy conclusions. Until the plan referred to is pursued, our knowledge of the connexions of parts of the brain and spinal cord must be imperfect; and the want of attention to it makes M. Foville's treatise incomplete.

Few of our readers, we think, will consider our remarks misplaced, concerning the value of the aid to be derived from comparative anatomy, when they learn the views propounded by the author. So great is the novelty of M. Foville's views, that many results previously received as established, are rendered doubtful by his examinations. For instance, among other novelties, he makes the following announcements: That the three divisions of each half of the spinal cord—viz. the anterior, middle, and posterior fasciculi—contribute almost equal portions to the cerebrum; and that the posterior fasciculus or restiform body, united by an offset to the cerebellum, may be traced through the cerebrum as the axis to which the nucleus of the hemisphere is attached, and may be followed to the anterior perforated spot, or "fundamental part" of the hemisphere. That the corpus callosum is derived chiefly from the same prolongation of the restiform body. That the hemispheres of the cerebrum are united by a commissure at the base, extending from the infundibulum to the apex of the pyramids, &c.*

This volume is, what it professes to be, a complete treatise on the anatomy of the brain and spinal cord, the centre of the cerebro-spinal system of nerves: it is preliminary to the drawing of conclusions respecting the function of these parts. For the views of the author on their physiology and pathology, we must wait for the publication of a succeeding volume. An active spirit of inquiry has evidently directed the investigations of the author: everything appears to have been personally examined. M. Foville states his views boldly, as a man who entertains no doubt of their accuracy; and we are sure that he wishes them to be examined carefully, and criticised with candour.

The author treats, first, of the anatomy of form of the spinal cord, cerebrum, and cerebellum; and then considers their structural anatomy. A dissertation on the origin of the cranial and spinal nerves follows next in order; and this portion of the book closes with the membranous investments of the cord and brain. An interesting inquiry is then entered into concerning the correspondence of the spine and cranium with the form of the inclosed masses, reference being made to the locality of the convolutions of the brain; and artificial regions are proposed, to determine with more precision the position of those convolutions. Accompanying the volume we have an atlas of twenty-three plates in lithography; the designs by Messrs. Beau and Bion, and the lithography by Artus. The delineations of the convolutions on the surface of the he-

* With all the candour that M. Foville has shown in his historical notice, we think he has scarcely given a due measure of praise to Reil, who certainly, as will be afterwards seen, preceded our author in many of his dissections of the cerebrum and cerebellum.

mispheres are excellent, but others of the medulla oblongata, &c. are very stiff, owing to the previous immersion in spirit of the parts from which they are taken. The execution of the lithography is good throughout. We must not, however, be misunderstood, as recommending the atlas to those who wish to learn the common opinions on the anatomy of the brain: the plates are designed to explain the views of the author, not to serve to instruct the beginner in his studies.

The spinal cord. The following remarks comprise the chief of M. Foville's views on this portion of the nervous centre. The three divisions of each half of the cord—viz. the anterior, middle, and posterior fasciculi, extending the whole length—increase in size superiorly, and constitute the medulla oblongata. In certain spots they differ in volume, the enlargement of the cord being occasioned by the greater size of one or other. Thus, in the cervical enlargement, from which the nerves of the cervical plexus are derived, the posterior and lateral fasciculi are the largest; in the brachial it is the posterior fasciculus that preponderates; whilst the greater size of that part of the cord, to which the lumbar nerves are attached, depends upon an increase in the lateral fasciculus. Superiorly, the lateral fasciculus increases in size. To it, on close inspection, may be seen attached along the line of the anterior roots of the nerves, "a great number of finer roots, proceeding from the contiguous part of the lateral fasciculus, and uniting with the former."* In the fetus it is said this lateral division of the cord is divided unequally into two by a gray semitransparent line on the surface; the smallest portion, called "accessory," is directed to the cerebellum, but the larger, the continuation, reaches the cerebrum. Fissures have been supposed to occupy the lines of attachment of the roots of the nerves, marking the limits of the fasciculi or divisions of each half of the cord; but M. Foville says there is not any fissure exteriorly in those spots, and that the roots of the nerves enter the cord by small apertures in a thin medullary fibrous layer on the surface. When this layer is torn through the fissures are exposed. For a detailed account of the minute structure of the cord we refer the reader to the work, p. 281. The attachments of the nerves will be noticed hereafter.

The commissural or connecting piece of the halves of the cord is seen by opening the anterior and posterior median fissures. It consists of a central gray part, covered before and behind by a medullary layer. This central part M. Foville considers the commissural part of the cord, that combines in action the opposite sides of the body. The layer of medullary substance in the posterior fissure is the posterior commissure, which serves as a bond of union between the posterior fasciculi. The stronger layer in the anterior fissure is the anterior commissure of the cord; it is "formed by a layer of white substance, hollowed in its whole length by very numerous small transverse fissures, very near one another, but which, it is very remarkable, exist only on the sides of that commissure, without encroaching upon the middle line, which presents all along a

* In a subsequent note at p. 530, the author throws some doubt on this statement. He says: "I have not been able to repeat my inquiries on this point frequently enough to convince myself of the existence of lateral nerves in the lumbar enlargement of the cord."

small crest or kind of very slightly-marked *raphé*." A depression is also seen on each side, and, when the fissure is widely opened, the transverse surface of the commissure, which passes from the one to the other half of the spinal marrow. Where the cord ends above in the medulla oblongata other fibres are seen to cross the anterior fissure, and connect the opposite halves of the cord: this appearance, known by the term *decussation of the pyramids*, is considered by M. Foville as but a part of the great anterior commissure, with its fibres more strongly marked and more evidently digitating. The author supports his views of the importance of this connecting portion of the cord, by the following remarks, which we extract:

"But what is the texture of this anterior commissure? Does it consist solely of fibrous laminæ, extended from right to left, to unite the two halves of the organ to which it belongs? Or does it, on the contrary, serve the purpose of a decussation of the one side with the other? This last opinion has been often suggested by the inspection of the parts, and not less often refuted by a physiological objection—the absence of all signs of decussation in injuries of the cord. Nevertheless, the question does not appear to me definitively decided. Firstly, the simple inspection of the anterior commissure is calculated to suggest the idea of a decussation between the two halves. Secondly, if this decussation exist, it should be between the anterior fasciculi, which unite so freely with that commissure; and an attentive inspection of those fasciculi seems to me to favour the opinion that they decussate, without interruption, from the top to the bottom of the cord." "Another reason which would lead us to admit the decussation of the anterior fasciculi of opposite sides of the cord, through the means of the anterior commissure, is the following: It is not uncommon to see the anterior fasciculi divided, at the top of the cord, into two or three portions. If the most internal of those portions is followed attentively, it is seen, after a course of greater or less extent, to end at the border of the anterior median fissure, where it terminates in a point, which penetrates obliquely into the corresponding wall of that fissure. Does not all this render probable the decussation between the anterior fasciculi of the spinal cord? But to appreciate the force of these reasons it is first necessary to consider the arrangement of the decussation of the pyramids, admitted by the majority of anatomists. We shall see further on that this decussation of the pyramids is but the continuance of a system of decussations, which commences between the cerebral peduncles, behind the corpora mamillaria, and is prolonged uninterruptedly to the pyramids." "Finally, all conjecture cannot supply the place of demonstration. The microscope seemed designed to render here a new service to science. Dr. Gruby has readily undertaken, at my request, the examination of those parts in which I suspected decussations. He has seen them (decussations), has made me see them; and he is moreover occupied in delineating them, to illustrate a description in which he is engaged." (p. 294-7.)

Following the order observed by the author, we take next the upper part of the cord or the medulla oblongata, the pons, and peduncles or crura of the cerebrum; all which intervene between the spinal cord and the hemispheres of the brain. To the anatomist and physiologist the investigation of these parts is most necessary and interesting, because of the present uncertainty of opinion respecting the disposition of the fibres, or the origin of the important nerves connected to them. We must confess we looked most anxiously through the pages containing the description of the above-named parts, and hoped to find (not that we in the least undervalue additional research,) announcements corroborative of our commonly received opinions, so that we might presume with something like confidence that we possessed a standard of knowledge. Our disappointment may be judged of when it is said, that the dissections of Foville unsettle our former supposed foundations, and render necessary farther examination. Much, certainly, do we wish that some one equal to the task, whose skill and accuracy are unquestionable, would enter on this difficult field of inquiry. In opposition to the received opinions, M. Foville says that all three fasciculi or divisions of each half of the spinal cord are continued to the cerebrum, the posterior or restiform body supplying only an offset to the cerebellum; that each, in passing the pons, contributes transverse fibres to its formation; and that the prolongations of the three, placed side by side, constitute the peduncle of the cerebrum. In this difficult and disputed region of anatomy we could wish more unexceptionable and illustrative plates than the author has given us, of the course in the brain of the prolongations from the three fasciculi of the spinal cord. The method we shall take is to follow uninterruptedly through the medulla oblongata, pons, and peduncle, the fibres continuous with either the posterior, anterior, or middle division of the cord, till they are about to spread out in the great or little brain: their distribution in the hemisphere will be subsequently mentioned.

*The medulla oblongata, pons, and peduncle of the cerebrum.* In these parts, placed in a line, in the order mentioned, are recognized continuations of the three fasciculi that constitute each half of the spinal cord. But the simplicity observable in the cord is much interfered with in the medulla oblongata.* Occupying the middle line in their whole extent is a commissure corresponding to the anterior of the cord.

The following is the best digest we could make of the author's opinions.

The posterior fasciculus of the spinal cord, known by the name of restiform body in the medulla oblongata, joined by the "accessory" portion of the lateral fasciculus, gives, at the lower border of the pons, some superficial transverse or commissural fibres to constitute the lower part of the pons, others appearing as a fibrous band on the surface between the fifth nerve and the portio dura of the seventh pair. Afterwards the restiform body divides into a posterior or cerebellar, and an anterior or cerebral portion. The posterior constitutes the inferior peduncle (Reil) of the cerebellum. The anterior or prolongation to the cerebrum, placed internal to the transverse fibres from the cerebellum to the pons, is then found to form successively the lateral boundary of the fourth ventricle and aqueduct of Sylvius, the inner part of the optic thalamus, and, finally, a portion of the lamina cribrosa, or *substantia perforata antica,* at the inner part of the fissure of Sylvius. Between the lower and upper borders of the pons, retrograde fibres are given from this fasciculus to the auditory and fifth nerves, also others deeper and transverse to the upper border of the pons. Beyond the pons this prolongation forms the part of the crus cerebri above the external depression, or, as Foville expresses it, "In other words, with the exception of some fibrous layers which descend into the lateral or the anterior fasciculus, all seen in profile be-

* M. Foville considers the peduncles of the cerebrum but one part, "*le tronçon pédonculaire,*" which consists of a right and left portion, united across the posterior perforated spot by decussating fibres.

hind the fasciculated region (lower half) of the cerebral peduncle, is continuous with the posterior fasciculus of the spinal cord."

The lateral fasciculus consists of two parts, as before said ; the small "accessory" being directed backwards beneath the superficial roots of the auditory nerve, and the other continued upwards to the cerebrum. It is in newly born infants that the accessory portion is seen. The course of the fibres of the cerebral portion is by the pons, where they are placed above the transverse fibres, and through the crus cerebri, as high as to the corpora albicantia. At the last spot the fibres end in an enlargement, resembling the half of the head of a club-stick slightly curved, from which fibres radiate to the cerebrum. In the crus they occupy a position between the prolongations from the posterior and anterior fasciculi. Opposite the lower half of the pons a large bundle is given to the deep transverse fibres of that body; and opposite to the tubercula quadrigemina, the *processus e cerebello ad testes* (superior peduncle—Reil,) joins the lateral fasciculus, and the offset from the tubercles to the olivary body traverses the fibres. Internally the fibres take part in a commissure to be presently noticed.

The anterior fasciculus, continued upwards as the anterior pyramid of the medulla oblongata, is described by anatomists as passing through the pons, and receiving in it additional fibres, to which are due its enlargement and conical form. But M. Foville states that this view rests on a theory of Gall, and is not supported by an examination of the structure of the parts. He attempts to show that the fibres are not added, but that the conical form results from fibres ceasing, from the base of the brain to the pyramid, along the middle line. In the crus, the prolongation from the anterior fasciculus of the cord occupies the lower or fasciculated region (the part below the depression on the outer side), whilst above it are the continuations of the posterior and lateral fasciculi, the last separated by the locus niger of Soemmerring. The following is the disposition of the fibres in their course from the hemisphere to the cord. In the crus they are inclined obliquely inwards from the hemisphere, the most internal ending in a commissure that occupies the middle line of the crura. The fibres that enter the pons, also decreasing in number from the upper to the lower border, are directed inwards, sometimes minutely divided, to the same median commissure, and assist in forming the transverse fibres of the pons. In the medulla oblongata the fibres join in the middle line, by means of the decussation of the pyramids, with the opposite half of the cord. So the number of fibres decreases downwards in the crus, pons, and pyramid, the strength of the intermediate commissure diminishing in the same degree.

Of much importance, in the opinion of the author, is a decussation at the base of the brain, or the median commissure that unites the "opposite halves of that portion of the axis of the nervous system comprised between the base of the cerebrum and the union of the medulla oblongata with the spinal marrow." It extends from the decussation of the pyramids to the infundibulum at the base of the brain, being, as it were, a continuation upwards of the great median commissure between the halves of the cord. Opposite the upper border of the pons it is most marked, and diminishes in strength from that point both upwards and downwards. To expose the commissure, take the base of a brain after the cerebrum and cerebellum have been divided so as to bring into view the third and fourth ventricles, and attempt the separation of opposite sides in the middle line of the fourth ventricle. Proceeding carefully, the separation is easy between the lateral fasciculi in the medulla oblongata, but impossible opposite the thickest parts of the pons and the fasciculated region of the crura cerebri. Between the lateral fasciculi the separated vertical surfaces are somewhat smooth, and present prominences with intervening depressions, the projections of one side fitting into the pits of the other. In the region of the crura cerebri is found a complete decussation, the fibres of one peduncle—say from the right—passing to the left, and *vice versâ*.* After decussating in the middle line, the fasciculated fibres of the crus cerebri are continued into the lateral fasciculus of the side opposite to that from which they proceed, resembling the decussation of the pyramids across the median fissure of the cord. What takes place in the pyramids is repeated in the whole length of the commissure, that is to say, from the summit of the pyramid to the infundibulum, except that the fibres opposite the pyramids are stronger than the remainder. From what has been said, the destination of the fibres in the crus cerebri may be understood: the oblique fibres on the under surface of the crus cerebri, directed to the middle line, are continued into the fibres of the lateral fasciculus of the opposite crus; whilst the more external fibres of the same surface, that reach inferiorly to the decussation of the pyramids, are connected with the anterior fasciculus of the cord of the same side, as well as with the lateral fasciculus of the opposite side.†

The author terminates the anatomy of the medulla oblongata, pons, and peduncle, with the following summary of the whole:

"In conclusion, three longitudinal fasciculi—viz. the posterior, lateral, and anterior of the cord—are found in the portion of the nervous centre that joins the spinal cord to the brain; the last having preserved its anterior position, the posterior having become external, and the lateral fasciculus posterior to the level of the floor of the ventricle of the cerebellum. A superficial part of the lateral fasciculus passes into the cerebellum.

"Some fibres disposed in transverse arches are also met with. The fibres with this direction are not alone those of the pons; to the same order belong the arciform fibres of Rolando, disposed in arcs of circles upon the side of the medulla,

* Here is an instance in support of the assertion, that M. Foville passes over Reil's merits. With this extract, and a future reference, it will be seen that Reil has completely noticed the appearances, though he does not state that the fibres on the under surface of the crus are derived from the prolongation of the lateral fasciculus of the opposite side. Reil, in treating of the fourth ventricle and the large bundles of the lateral fasciculi in the floor, says: "One other layer—the vertical fasciculi—composed, not of nervous matter alone, but of cellular membrane likewise, and vessels in unusual proportion, as may be conjectured from its toughness, extends from behind the pyramids, behind the upper transverse fibres of the annular protuberance, and below the ansa of the anterior peduncles of the cerebellum, to the gray substance which is found between the crura cerebri, extending beyond the corpora albicantia; thus building the floor of the third ventricle, and becoming continuous with the infundibulum. The fibres of this structure seem in some degree to decussate each other." (Mayo's translation of Reil's Commentaries. Part ii.)

† *Note of the author.* "This system of decussations, suspected by Rolando, who denied that of the summit of the pyramids, has been partly seen by Valentin, and recognized by M. Longet, to whom we had the pleasure of communicating it. No author hitherto has represented it altogether as it is described in this work, and has been made known some years by us to the Academy of Sciences and of Medicine."

the concavity above, which join the pyramid and olivary to the restiform body, and contribute to the origin of many nerves. A considerable commissure decreasing from above. Lastly, two layers of fibres occupy the vertical median surface.

"The posterior fasciculus, become for a time external, is prolonged under the tubercula quadrigemina, the optic thalamus, and the perforated quadrilateral space. A considerable part of each fasciculus enters the cerebellum, where it will soon be followed. Other offsets from this fasciculus belong to the transverse fibres of the pons.

"The anterior fasciculi unite the anterior parts of the cord with the centre of the base of the cerebrum, into which they penetrate by a sort of ring, formed by the posterior fasciculus united with the optic thalamus and the perforated quadrilateral spot.

"The lateral fasciculus, continued into the hollow of the crus cerebri above the locus niger, does not cease altogether in that spot; its prolongation into the cerebrum will be afterwards described.

"The transverse fibres (of the pons) are offsets from the posterior, lateral, and anterior fasciculi. They combine with all those fasciculi, also with the portion of the peduncle of the cerebellum annexed to the protuberance. We shall return to the transverse fibres of the cerebellum. It suffices here to point out that they contribute to form the ring of the protuberance.

"The commissure contains decussating fibres proceeding, in great part, from the fasciculated region of the crus. These fibres enter, after their decussation, the median vertical surfaces; those from the right crus appertain to the vertical surface of the left side, and reciprocally, those from the left crus proceed to the vertical fibres attached to the right lateral fasciculus." (p. 325.)

After an attentive consideration of the views of the author, let us pass in review his statements respecting the anatomy of the portion of the nervous centre between the hemispheres of the brain and the spinal cord. Regarding this part of the nervous system as the most important, and considering a correct knowledge of its anatomy necessary to the understanding the dependence of the various portions of the brain, it behoves us to receive with great caution all doctrines that introduce great changes. It is in this spot that the fibres seem to be rearranged before being developed into the hemispheres and convolutions, so that in all examinations of the brain it would appear necessary to start from it as from a centre or link between the cord and brain. As M. Foville's statements are supposed to be founded on observation, it must appear rash to give an opinion opposite to that of the author, unless the same is based upon facts obtained by dissection. Where it will be necessary to differ we shall not dissent without much hesitation, even though our opinion is supported by other anatomists and experience.

According to Foville, the posterior fasciculus of the cord (restiform body) enters as largely into the structure of the cerebrum as into that of the cerebellum. If the matter is examined anatomically, it appears that the restiform body is connected to the pons, giving probably a few superficial fibres to it, as described by Tiedemann, and ends in the inferior peduncle of the cerebellum, as so ably traced by Reil. Foville's assertion can, in our opinion, be explained only by presuming that some of the fibres of the lateral fasciculus, which spread out in the floor of the fourth ventricle, and are closely connected with the restiform body, have been traced to the cerebrum as a prolongation from the posterior fasciculus. That this is the explanation of the origin of those fibres followed to the hemisphere is rendered more probable by the statement of the author, that, with the exception of a few fibres, "all the part of the crus cerebri, seen in profile above the groove on the outer side," belongs to the prolongation from the posterior fasciculus. Now it must be so evident to all, that the portion of the crus referred to is formed by the superior peduncle of the cerebellum or prolongation from the cerebellum to the cerebrum, that we should infer some oversight, did not the author consider the superior peduncle of the cerebellum to perforate the prolongation of the posterior fasciculus, and join the lateral fasciculus. It is easy, in the dissection of hardened brain, to detach the superior peduncle of the cerebellum from the prolongation of the lateral fasciculus on which it lies, and to follow it backwards to the cerebellum or forwards to the optic thalamus in which it expands, as described by Reil; and the knowledge thus obtained is opposed to Foville's view of the peduncle perforating the posterior fasciculus, and joining the fibres of the prolongation of the lateral fasciculus opposite the tubercula quadrigemina.

The lateral fasciculus (made of comparatively little importance in the production of the cerebrum, since it is said to end at the upper part of the crus in a rounded head, from which few fibres again radiate, but without direct continuation,) appears to be limited in lateral extent by the author, and for the reason above mentioned, viz. the assigning a portion of its fibres to the posterior fasciculus. The fibres are described as uniting in a commissure in the middle line, which is strongest opposite the upper border of the pons. The arrangement of the commissural surfaces was described by Reil, without his naming the appearance, a commissure. We noticed before in a note that Reil observed the extent and disposition of the vertical fibres, and we give now an extract from the same author, to show that he was also acquainted with those fibres that cross in the middle line. He describes them as resulting from a union of the superior peduncles of the cerebellum. He says: "The peduncles now plunge downwards, forwards, and inwards into the cylinder, (the portion of the crus cerebri above the locus niger,) having above the tubercula quadrigemina; above and without, the fillets; within, the vertical fasciculi; below the latter they unite by means of an *ansa*, which is several lines in thickness, and forms the upper wall of the foramen cæcum; it is a question whether a complete continuity or [an ?] anastomosis occurs here." We have examined the commissure according to the method recommended by Foville, but are inclined to the opinion that Reil's description is correct, viz., that at the posterior part, where the surfaces are vertical, it is a question whether there is an interchange of the fibres of opposite sides; and that the fibres, crossing the middle line, are part of the superior peduncle of the cerebellum, forming the "ansa" of that author, and not fibres from the prolongation of the lateral fasciculus whilst contained in the crus cerebri.

Our experience is likewise opposed to the opinion of Foville, that the most internal fibres of the under surface of the crus, directed obliquely inwards and continuous with the anterior fasciculus of the cord, are derived by decussation from the crus of the opposite side. An examination demonstrates that those fibres do not cross the locus perforatus in the middle line, but wind backwards, round the inner part of the crus, to the projection in the floor of the fourth ventricle, remaining on the same side of the body.

In figure 1 of plate v, the pons is represented as having three distinct canals in the transverse fibres, to allow the passage of the prolongations of the three fasciculi of the cord. Those are said to be produced by the transverse fibres continued from each fasciculus to the pons; but the appearance is rather singular, when it is remembered that the prolongation of the lateral fasciculus is, and that of the posterior would be above or behind the transverse fibres from the cerebellum to the pons.

THE CEREBRUM. Whilst following the author through his investigations of the anatomy of the brain, we shall select only those parts that are considered by him of greater consequence. With the general form and divisions, or the external anatomy, there is little to interest, except the classification of the convolutions. Much importance is given by the author to the fissure of Sylvius, and the perforated spot at its inner termination. These last two are considered "fundamental" parts in the human brain, and as the greater or less development of them distinguish the brain of man from that of beings lower in the scale, their examination cannot be void of interest.

*Convolutions.* Since the assertion of Gall, that the different faculties of the mind are located in the convolutions of the cerebrum, and that, according to the condition of these manifested by the form of the exterior of the skull, the mental endowments of an individual can be recognized, much attention has been very justly given to the study of the form of the brain. So weighty a subject has not escaped our author, and the opinions of one who has so extensively studied the brain deserve our most attentive consideration. What is advanced by the author has been obtained from observation of the brain, and is the more worthy of credit, because it is not brought forward by a partisan in support of a particular theory; for there seems a greater desire to record facts than to gain any triumph over an opponent. The author is not favorable to the opinion of the convolutions on the surface of the brain being either very numerous, or similar on the opposite hemispheres. Nor does he consider them always proportioned to the size of the brain; the extent of surface being sometimes inversely as the mass of the hemisphere. He asserts that the idea of the possibility of the convolutions being "limited, named, and traced on the surface of the cranium by a sort of chart," is altogether opposed to anatomical investigation. "There are long lines of convolutions uninterrupted on the surface of the brain, groups distinct from other groups; but these lines and groups merge in many points into the contiguous parts by direct union of the convolutions." Analogy, moreover, is opposed to the supposition that a single part serves to constitute a number of different organs, for it is found that a combination of different parts is necessary to produce one organ. The convolutions, too, are folds of one membrane, the cortical substance, which is plicated like the processes of a serous membrane, and is continuous like it over the surface. In many animals there are no convolutions; and in a hydrocephalic head, the convolutions may be unfolded by the fluid, the exterior presenting a smooth surface. Into the question of phrenology the author does not enter fully in this volume; but he considers its accuracy doubtful in the present state of our knowledge. An attempt is made to arrange the convolutions in four classes, and to ascertain not only their composition and dependence on the deeper structures, but their correspondence to certain known parts of the skull. To accomplish the latter purpose, the skull is marked into regions with great care and minuteness; but we must refer those who wish to ascertain the facts bearing on phrenology to an examination of the work. We cannot do more than give an abridgment of the writer's answer to the question: Does the form of the cranium point out the form of the brain? Only in a general way, viz. supposing the brain and skull natural, a long cranium cannot contain a short brain, nor a short skull a long brain. The same may be said of the general form of both; but in proportion as we inquire into the correspondence of individual convolutions with their containing case, so is precision and certainty destroyed. Thus a large forehead shows only a large frontal development of the cerebrum. Indeed the variable thickness of the skull, and the difference in the size of the frontal sinus, prevent the accurate determination of the size or outline of the underlying parts of the brain. In two individuals who have the same prominence and size of forehead, the same thickness of skin and bone, the same depth of frontal sinus, the dimensions of the frontal lobes may be very different. Foville says farther: "I do not doubt the possibility of ascertaining, with some degree of accuracy, by an examination of the skull during life, the relative development of the principal regions of the brain: I go farther, and think that, within certain limits, the physiological signification of the forms of the human head can be determined. But that science (phrenology) is not established, and it can be safely based only on exact anatomical knowledge." This opinion of Foville, who has practically investigated the subject, will, we trust, induce others to enter the same field of labour. We have long felt the views of phrenologists to be imperfect, because they have been supported chiefly by pathological facts and ingenious argument, instead of being founded on anatomical knowledge. Are not conclusions as to the functions of the brain, deduced from an examination chiefly of the skull, likely to be fallacious, when so little is certainly known of the contained organ, as regards either the position and extent of the convolutions, or the precise connexions of the component parts? Till those facts are ascertained, we cannot hope for any accurate acquaintance with the organ of the mind, and we would, therefore, urge upon inquirers to make the brain rather than the skull the source from which to obtain knowledge.

These are the four orders of convolutions in each hemisphere distinguishable one from another:

1. In the first order there is but a single convolution, the convolution of the band* (*circonvolution de l'ourlet*) or convolution of the corpus callosum of Reil. It surrounds the hemisphere like a riband, is fixed by each end to the perforated spot, and is divided into a large or vertical and a small or horizontal portion. The vertical portion commencing at the front of the perforated spot, and supporting the olfactory nerve, turns upwards round the front of the corpus callosum; placed on the upper surface of this body it bends round the posterior fold, and finally skirts along the crus cerebri, giving rise to part of the fissure of Bichat, to end

* Beneath the convolution is a small band of white fibres, similar to a hem in the border of a cloth; hence the name of convolution of the hem or band. The same is the covered band of Reil.

in the same perforated spot. The horizontal portion, situate over a small fibrous arch (*cintre fibreux*), is attached in its whole length to the outer part of the perforated spot, and unites the extremities of the vertical portion. To the outer border of this portion are connected the convolutions of the island of Reil, and that of the olfactory nerve.

2. The convolutions of the second order are two: one, the largest, occupies the circumference of the hemisphere; the other surrounds the island in the fissure of Sylvius. The largest commences at the base of the brain, where it is fixed to the horizontal portion of the convolution of the first order, and is internal to the groove for the olfactory nerve; at the front of the brain it turns upwards to the highest part of the hemisphere, separating the outer from the inner surface, courses round the posterior lobe, and ends in the inferior, between the convolution of the band and that of the fissure of Sylvius. The remaining convolution, connected at its origin to the other, surrounds the fissure of Sylvius, giving rise to three lips or borders, occasioned by two considerable indentations; it is united to the horizontal part of the convolution of the band by the gray substance of the island of Reil.

3. The third order of convolutions unites the first and second orders; so that some pass from the great portion of the convolution of the band to the large convolution of the second order, and others, from the horizontal or attached portion of the same convolution to that of the fissure of Sylvius. The first set are seen on the inner surface of the hemisphere, and on the posterior and inferior lobes. The other set form the island of Reil, and occupy the fissure of Sylvius.

4. To the fourth order belong all the convolutions on the outer surface of the hemisphere and under part of the anterior lobe; they intervene between the two convolutions of the second order, that is to say, between the one of the fissure of Sylvius and that separating in the middle line the inner from the outer surface of the hemisphere. These convolutions will increase or diminish in length as the convolutions they connect approach at their commencement and termination; some are divided in their course. For a full description consult the work, p. 217, et seq.

From what has been said of the four orders of convolutions, it will be seen that only the convolution of the first order is united directly to the perforated spot; those of the second order being joined to the horizontal or attached part of the first, whilst those of the remaining two orders do not reach that spot. It is this spot that Foville considers the fundamental part of the brain,—the source of the convolutions and certain parts of the cerebrum, the point of termination of the prolongation to the cerebrum of the posterior fasciculus of the cord, and from which arise the olfactory and optic nerves. To support what we should call a theory, the author shows an anxiety which is not commendable. Accordingly, though the third and fourth orders of convolutions do not reach the perforated spot, as the third is connected to the first order, and the fourth "may be considered a sort of prolongation from the convolutions of the third order," they must, he thinks, be allowed indirectly to reach it. Such reasoning, if allowed to be substituted for that demonstrative evidence that the examination of the brain requires, will be most likely to lead into error, and to make of little value any of the conclusions. Much good will no doubt arise from a classification of the convolutions; for though we are not prepared to pronounce perfect the plan followed, the attempt will serve to point the direction of future inquiry, and assist in other arrangements.

*Fissure of Sylvius.* A minuteness of description is the chief novelty of this part. The fissure occupies, as is well known, a transverse position at the base of the brain, from which it is continued upwards and backwards, and it is remarkable in circumscribing the convolutions of the island. Narrow at its inner termination, where it presents the perforated spot, it enlarges, and finally ends in three borders or lips, a superior, anterior, and posterior, which are folds of the convolution of the second order before noticed. Corresponding to the space inclosed by the lips of the fissure, or to the island, is the nucleus of the hemisphere, viz., the corpus striatum and optic thalamus, situate on the crus cerebri. In the human brain the tip of the posterior border reaches in front of the anterior. In no other brain does the posterior lip pass the limits of the anterior; and in proportion as the animal is higher or lower in the scale does the convolution approach to a complete circle or recede from the same.

The *perforated quadrilateral spot** (*espace ou quadrilatère perforé*) is considered by Foville the fundamental part of the cerebrum; and to it, as will be seen, are connected many important structures of the hemisphere. According to the views and description of the author, this space has been hitherto much neglected by anatomists. Its situation is at the inner part of the fissure of Sylvius, anterior to the crus cerebri. To it the olfactory and optic nerves are connected, and from it seem to radiate, as from a centre, the convolutions of the surface of the brain. The limits of the space are, the optic tract behind; in front, the convolution with which the roots of the olfactory nerve unite; internally, the border of the hemisphere; and externally, the small or horizontal portion of the convolution of the band. Crossing from the outer to the inner part of the space is a whitish layer† which divides it into two, gray matter being found both before and behind it. The collection of gray matter behind is connected with the commissure of the optic nerves, closing there an interval, and is analogous to the gray root of the olfactory nerve; the gray matter in front of that band joins the roots of the olfactory nerve. The proportion between the size of the perforated spot and the surrounding convolutions marks the degree of importance of the brain. Thus the more the brain enlarges and swells laterally around the peduncle, the more the perforated spot is hidden, until in man it is the roof of a cavity at the base of the brain. As the cerebrum decreases laterally, the spot becomes superficial, and may equal in size the base of the hemisphere. The same inverse proportion is manifested between the perforated spot and the island of Reil; the one enlarging as the other diminishes. In this portion of the hemisphere terminates the prolongation from the posterior fasciculus of the spinal cord; but its consequence as an elementary part of the brain will be referred to when treating of the structure.

*Structure of the cerebrum.* The cerebrum consists, for the most part,

* *Substantia perforata antica* of anatomical works; *lamina cribrosa* of Reil; and *espace perforé* of Vicq d'Azyr.

† This is described by Reil as the fillet of the lamina cribrosa. (Op. cit.)

of fibres that are directed from the centre to the convolutions on the surface; from the exterior to the middle line; or from the front to the back of the brain. Two chief sets of fibres have been considered to constitute the bulk of the brain: One, the diverging system, representing a cone (fibrous cone of Reil), whose apex is in the crus cerebri, and base at the circumference of the hemisphere. The other, the converging or commissural system, of which the corpus callosum and anterior commissure are the principal, united in the middle line, to combine in one whole the opposite halves of the brain. Foville undertakes to show that this view is incorrect. He asserts that only some of the convolutions receive diverging fibres from the peduncle, the others being supplied from the circular fibrous band* (*ruban fibreux de l'ourlet*) beneath the convolution of the band, which surrounds the root of the hemisphere. He also adopts the view, which considers the corpus callosum derived from the base of the brain and parts constituting the nucleus of the hemisphere, instead of from the convolutions on the surface. In the description of the convolutions, it was stated that the first and third orders were united, and that the second and fourth formed another system. Now, it is to the second and fourth orders on the outer surface of the brain that the diverging fibres of the peduncle reach; whilst to the first and third are continued fibres from the fibrous circular band around the root of the hemisphere. Let us trace shortly the arrangement of each set of fibres.

*Peduncular* or *radiating fibres.* In our notice of the crus cerebri, the prolongations to the cerebrum of the three fasciculi of the cord were left to be afterwards taken up. Before tracing these farther, it may be well to premise that each hemisphere consists of a central part or nucleus, the continuation of the cord, and of an enveloping portion connected to the other by fibres. Through the central part, consisting of the corpus striatum and optic thalamus, course the fibres of the crus or peduncle, dividing those two bodies into an upper or ventricular, and a lower or extra-ventricular portion.

The continuation of the posterior fasciculus of the cord, forming the upper part of the crus cerebri, is prolonged forwards by the side of the aqueduct of Sylvius, giving rise to the roof of the aqueduct and to the posterior commissure, and bifurcates opposite the optic thalamus. One portion, the radiating, passes through the thalamus, gives fibres to the optic nerve and corpus callosum, and ends at the outer border by joining the radiating fibres of the middle fasciculus. The remaining part (*partie centripète*) is continued along the third ventricle to the anterior commissure, where it likewise divides, an offset ascending to the lateral ventricle as the tænia semicircularis, the continuation entering the perforated spot below the commissure, joins the fibrous arch (*cintre fibreux*) of that region, and gives attachment in front to the olfactory and behind to the optic nerve. This centripetal portion of the posterior fasciculus is a sort of axis to which the nucleus of the hemisphere is attached; for the corpora quadrigemina, optic thalamus, tænia semicircularis, and corpus striatum, receive prolongations from it.

The portion from the middle fasciculus, occupying in the crus a position intermediate between that from the anterior and the posterior, is

* Covered band of Reil.

joined by the superior peduncle of the cerebellum, and ends, as before said, in a rounded projection near the optic thalamus. From this prominence arise other fibres, of which a few turn forwards to the perforated spot, the remainder enter the nucleus, and emerging on the outer side, join the radiating set to the convolutions.

The prolongation of the anterior fasciculus, stronger than the others, radiates to the outer side of the nucleus, the fibres being arranged like the half-opened tail of a peacock, with the concavity below. This appearance is named by the author fibrous fan (*éventail fibreux*). On the convexity of the fibres are the upper or ventricular masses of the striate body and optic thalamus, and in the concavity are placed the extra-ventricular portions of the same bodies.

The disposition of the fibres of each fasciculus having been followed into the nuclear bodies, it remains only to observe that they radiate from the outer surface of the same. Being arranged in layers they extend to the circumference of the hemisphere, and enter the convolutions on the outer surface, or those of the second and fourth orders. Near the nucleus, fibres to the corpus callosum are transmitted through intervals between them.

*Circular fibres.* All the convolutions of the first and third orders, or those on the inner surface of the hemisphere, and in the fissure of Sylvius, are attached to the circular instead of the diverging system of fibres. The source of these fibres is the band* (*ruban fibreux*) beneath the convolution of the same name. Like the convolution, the fibrous band consists of two parts, a free or vertical, and an attached or horizontal. From the free portion surrounding the root of the hemisphere, are derived offsets to the convolution covering the band, and to those on the inner surface of the brain. The convolutions of the island of Reil, contained in the fissure of Sylvius, receive their fibres from the attached portion of the band, which represents a fibrous arch (*cintre fibreux*), situate along the outer side of the perforated spot, and corresponds to the outer root of the olfactory nerve. Reserving for the present the notice of this arch, it will be sufficient to observe that from its outer side radiates the layer of fibres for the convolutions of the island.

*Perforated spot.* The author was very minute in his description of the boundaries, form, &c. of this spot, nor is he less particular in the enumeration of the various structures that enter into it. After the removal of the optic nerve, and the gray substance attaching its commissure to the prolongation (*partie centripète*) of the restiform body, the under-mentioned structures will be found in this portion of the hemisphere. Firstly,† occupying about the centre of the space, the prolongation (*arête centripète*) from the posterior fasciculus of the cord, which is directed outwards to the fibrous arch (*cintre fibreux*) on the outer boundary; to it are united in front the olfactory, and behind the optic nerve. Beneath the first layer is a prolongation, with a similar direction, from the middle fasciculus of the cord. Still deeper is the anterior commissure, with the extra-ventricular mass of the corpus striatum in front, and that of the optic thalamus behind it. Placed on the outer

* It was described by Vicq d'Azyr.

† The base of the brain being upwards.

side of the spot, corresponding to the attached part of the convolution of the band, is the fibrous arch (*cintre fibreux*) named olfactory arch by Rolando. Giving attachment to the olfactory nerve, the arch joins by its extremities the fibrous band beneath the convolution before named. Internally, it gives attachment to the prolongation of the posterior fasciculus of the cord, and to the anterior commissure; and externally radiate from it two layers of fibres, one ascends round the outer surface of the corpus striatum, and optic thalamus to join the corpus callosum, the other separated from the former by a mass of gray matter, is destined to the convolutions of the island of Reil.

*Corpus callosum.* This body forms the fibrous covering of the cerebral nucleus, and, constituted of diverging fibres,* unites finally in the middle line. It receives fibres of origin from many sources. Firstly, from the fibrous arch on the outer side of the perforated spot. From it the fibres radiate, the anterior to the front, and the posterior to the back of the corpus callosum; but the central, united with fibres of the anterior commissure, ascend external to the central masses of the hemisphere, pass between the diverging or peduncular fibres, and end in the middle part of the corpus callosum.† Secondly, from the prolongation of the posterior fasciculus of the cord; these fibres pass through the corpus striatum and optic thalamus. Thirdly, from the tænia semicircularis, and the ventricular masses of the corpus striatum and optic thalamus. So that, except the origin from the fibrous arch, the other processes are continuous with the centripetal part of the posterior fasciculus. Where the fibres become horizontal, to build the roof of the lateral ventricle, the corpus callosum presents a rounded prominent border.

The following are the generalizations of the author on the prolongations of the fasciculi of the cord to the brain:

"All the free surfaces of the cerebral nucleus, viz., of the ventricles, of the perforated spot, of the extra-ventricular surface of the corpus callosum, are formed from fibrous layers, or gray masses attached to the cerebral prolongation of the posterior fasciculus.

"All the free surface of the hemisphere, that is to say, the surface of the convolutions, belongs to the cortical substance, into which is continued, contributing to its formation, offsets from the posterior fasciculus. . . . .

"The prolongations of the anterior and lateral fasciculi occupy always a deeper situation in the cerebrum. . . .

"If they are examined in either the cerebral nucleus or the hemisphere, they are always surrounded by the developments from the posterior fasciculus. They may approach the surface by their extreme ramifications, but they never expand in that surface.

"The cerebral prolongation of the posterior fasciculus occupies in the brain the same position as the skin and mucous membrane in the body; these being supplied by nerves from the posterior, never from the anterior fasciculus.

"The cerebral prolongations of the anterior fasciculus, contained in the interval of the membranous expansion of the posterior, occupies in the brain a position similar to that of the muscular system in the body, which is excited by nerves of the anterior fasciculus." (p. 488.)

* This view of the corpus callosum being derived from the radiating fibres of the crus cerebri, was held by Tiedemann.

† Reil states that the central fibres of the corpus callosum meet at an acute angle, and sometimes directly anastomose with the middle fasciculi of the fibrous cone, derived from the inner and outer walls of the capsule of the corpus striatum.

Still unable, notwithstanding an attentive perusal of the work, to entertain the opinion of the author, that a prolongation from the posterior fasciculus of the cord is the elementary part of the cerebrum, we must dissent from the conclusions deduced. By this declaration we do not intend it to be thought that M. Foville is supposed to describe fibres which have not been seen, but rather, to trace to the perforated spot a bundle of fibres belonging to the lateral, instead of the posterior fasciculus of the cord. It is much to be regretted that there is no good representation of the described prolongation (*partie centripète*) in its course in the cerebrum. In plate xviii is the best view, but even this is so far from satisfactory, that it might perhaps render doubtful rather than support the opinion of the author. If it is possible then, as suggested, that M. Foville has pursued in his dissections a portion of the lateral fasciculus, seen in the floor of the fourth ventricle, for a continuation of the restiform body all the offsets from the supposed posterior fasciculus would be given from the lateral fasciculus. In that case it would be needless to urge other objections, or to make further remark upon opinions, which would approach so nearly to those at present held. Nor does it so satisfactorily appear that the view of the anatomy of the corpus callosum is altogether correct. Suppose it true, as stated by Tiedemann, that the fibres of the corpus callosum are derived from those of the crus, neither this opinion, nor that of our author will, we think, explain all the appearances of that structure. The large mass of diverging fibres to the anterior and posterior lobes of the brain are independent of those that take origin from the penduncular fibres, and constitute the middle part of the corpus callosum. With Reil we are disposed to believe that the greater number of the fibres are derived from the surface of the brain, particularly the large anterior and posterior prolongations, but that other central fibres are connected with the diverging system of the crus or peduncle, and with the nucleus of the hemisphere. Though anxious to give our author all credit, we have felt it our duty to criticise his statements ; not however, for the purpose of finding fault, but with the wish of arriving at the truth, and stimulating others to inquiry and examination. The length and minuteness of the review will sufficiently mark our opinion of the value of the work.

ORIGIN OF NERVES. Our limits will not allow more than a mere sketch of this part of the volume: to follow the author in the origin of the cranial nerves, would demand not only much space, but much attention on the part of our readers; for here detail seems to be carried to its utmost limit. All who are anxious to be more particularly informed will consult the work, (p. 491, et seq.) The author rejects the usual classification of spinal and cranial nerves. Assuming that he has proved that the anterior, middle, and posterior fasciculi of the cord are continued by distinct portions into the cranial mass, he divides the nerves accordingly into three classes : nerves of the posterior, anterior, and middle fasciculi of the cord. Those that take origin from the spinal cord are named common, and those from the cranial mass, special nerves.

1. *Nerves of the posterior fasciculus of the cord.* In this class the nerves are connected to gray masses or ganglia. In the spinal cord the ganglia are common; in the skull the cerebellum is the ganglion of the auditory and fifth, and the cerebrum, of the optic and olfactory nerves.

In this division are comprised nerves attached to the posterior fasciculus whilst forming part of the spinal cord, medulla oblongata, cerebrum, and cerebellum.

*a.* From it in the spinal cord are the posterior roots of the spinal nerves, thirty-one in number, which are united to the superficial fibres of the posterior fasciculus, and to the gray substance of the cord.

*b.* From it in the medulla oblongata are only two, the pueumo-gastric and glosso-pharyngeal.* Soemmerring's deep origin from the gray matter at the posterior part of the bulb is adopted; and it is said that the nerves are joined by the arciform fibres.

*c.* From it in the cerebellum are derived the auditory nerve and large portion of the fifth. The auditory, attached to the outer side of the restiform body, has one prolongation to the floor of the fourth ventricle, and another backwards beneath the cortical substance of the cerebellum, which previously unites with a similar part of the fifth; the union of the two offsets gives rise to a membraniform layer, that lines the under surface of the cortical substance. The large portion of the fifth is fixed to the deeper parts of the restiform body; it gives backwards a layer to join with that of the auditory nerve, and forwards transverse fibres to the pons.†

*d.* From the same axis in the cerebrum are the olfactory and optic nerves. The optic is connected internally to the prolongation of the posterior fasciculus of the cord; externally, to the superficial part of the optic thalamus and the tubercula quadrigemina; and anteriorly, at the commissure, there is an attachment in front and behind to the gray matter of that part. The olfactory nerve is united to the surface of the perforated spot, to the prolongation of the posterior fasciculus of the cord to that spot, and to the gray substance of the hemisphere; still deeper, it is connected with the fibrous band and arch in relation with the convolution of the first order, to the extra-ventricular part of the corpus striatum and the fibrous layer covering it, and to the anterior commissure of the brain.

2. *Nerves of the anterior fasciculus of the cord.* This fasciculus extending upwards into the cerebrum, gives origin also to spinal and cranial nerves. None of the nerves of this class present ganglia. In the length of the cord, the nerves are united with those derived from the posterior fasciculus, but the remainder are separate.

*a.* From it in the spinal cord, are the posterior roots of the common spinal nerves, which plunge into the substance of the cord, some fibres uniting with the anterior fasciculus, others with the gray substance.

*b.* From it in the medulla oblongata spring the ninth and sixth nerves, and the portio dura. For the sixth and ninth the common origin is given; but the portio dura is traced beneath the transverse fibres of the pons to the prolongation of the anterior fasciculus.

*c.* From it in the crus cerebri is the third nerve, which arises from the fasciculated part of the crus, and from the locus niger of Soemmerring.

* The author includes these amongst the spinal nerves, though they are here separated.

† In fig. 3 of plate ii, fibres are delineated as joining the large portion of the fifth to the auditory nerve.

3. *Nerves of the lateral fasciculus of the cord.* This tract has attached to it both spinal and cranial nerves, but all are special and recognized by particular names.

*a.* To it in the spinal cord is attached but one nerve, the spinal accessory,—a portion of the eighth cranial nerve of Willis. This arises from the "accessory" portion of the lateral fasciculus, which is continued to the restiform body.

*b.* To it in the cranium three nerves are connected; a portion of the seventh pair, a part of the fifth, and the fourth nerve. The small part of the seventh (*pars accessoria* Wrisberg) is fixed to the lateral fasciculus at the pons. The masseteric nerve, a portion of the fifth, perforating the transverse fibres of the pons, is united to the lateral fasciculus. The fourth cranial nerve is united to the prolongation issuing from the posterior pair of the corpora quadrigemina to join the lateral fasciculus in the crus cerebri.

With the preceding analysis of this work we must be content to terminate our notice. We do not presume to have extracted from it all its stores, for we have confined ourselves to the consideration of the spinal cord and the cerebrum. Recommending strongly the work to the attentive perusal of all, we must repeat once more our desire that the views brought forward may be candidly but critically examined; and pleased shall we be to find a similar spirit of inquiry spreading its influence over every future attempt to enlarge our extent of knowledge of the brain. It has been our aim in this notice to review those parts which would give the anatomical opinions that were new and important, and tend to develop the physiological views of the author. We have therefore selected the cord and its continuations into the hemisphere of the cerebrum, and have traced the posterior fasciculus, through the medium of the perforated spot, into the convolutions on the surface of the brain. With this knowledge, it will be easy to comprehend some of the future views of the author shadowed forth in a concluding passage of his work:

"To form a physiological theory from this anatomy of the nervous system, it will suffice to give a direction to the course of the nervous agency. That direction is known. Departing from the peripheral parts of the body it reaches the ganglia and the posterior fasciculus of the nervous centre, which communicate with the interior and the exterior of the cranial mass. In that mass is a part, the cortical substance, which is intermediate between the termination of the posterior and the commencement of the anterior fasciculus. From the adherent surface of the cortical substance spring the anterior and lateral fasciculi of the spinal cord; and from those fasciculi are derived the nerves that excite the muscles." (p. 655.)

We must not conclude without offering to the excellent author, the homage of our highest respect for his talents, industry and perseverance; for the comprehensive views, liberality of sentiment, and honesty of purpose exhibited in his work; and, we must add, for the high honour, generosity, and philanthrophy, conspicuous in his conduct as a man and a physician.

